# Hyaline cartilage calcification of the first metatarsophalangeal joint is associated with osteoarthritis but independent of age and BMI

**DOI:** 10.1186/s12891-016-1324-0

**Published:** 2016-11-15

**Authors:** Jan Hubert, Thelonius Hawellek, Sandra Hischke, Jessica Bertrand, Matthias Krause, Klaus Püschel, Wolfgang Rüther, Andreas Niemeier

**Affiliations:** 1 Department of Orthopaedics, University Medical Center Hamburg-Eppendorf, Martinistraße 52, Hamburg, 20246 Germany; 2 Department of Medical Biometry and Epidemiology, University Medical Center Hamburg-Eppendorf, Martinistraße 52, Hamburg, 20246 Germany; 3 Institute of Experimental Musculoskeletal Medicine, University Hospital Münster, Domagkstrasse 3, Münster, 48149 Germany; 4 Department of Orthopaedic Surgery, Otto-von-Guericke University Magdeburg, Leipziger Str. 44, Magdeburg, 39120 Germany; 5 Department of Osteology and Biomechanics, University Medical Center Hamburg-Eppendorf, Lottestraße 59, Hamburg, 22529 Germany; 6 Department of Legal Medicine, University Medical Center Hamburg-Eppendorf, Martinistraße 52, Hamburg, 20246 Germany

**Keywords:** metatarsophalangeal joint, Hallux, Osteoarthritis, Cartilage calcification, Hyaline cartilage, Age, BMI

## Abstract

**Background:**

Hyaline cartilage calcification (CC) is associated with osteoarthritis (OA) in hip and knee joints. The first metatarsophalangeal joint (1^st^MTPJ) is frequently affected by OA, but it is unclear if CC occurs in the 1^st^MTPJ. The aim of the present study was to analyze the prevalence of CC of the 1^st^MTPJ in the general population by high-resolution digital contact radiography (DCR) and to determine its association with histological OA severity, age and body mass index (BMI).

**Methods:**

168 metatarsal heads of 84 donors (*n* = 47 male, *n* = 37 female; mean age 62.73 years, SD ±18.8, range 20–93) were analyzed by DCR for the presence of CC. Histological OA grade (hOA) by OARSI was analyzed in the central load-bearing zone of the first metatarsal head (1^st^ MH). Structural equation modeling (SEM) was performed to analyze the interrelationship between CC, hOA, age and BMI.

**Results:**

The prevalence of CC of 1^st^MH was 48.8 % (41/84) (95 %-CI [37.7 %, 60.0 %]), independent of the affected side (*p* = 0.42), gender (*p* = 0.41) and BMI (*p* = 0.51). The mean amount of CC of one MH correlated significantly with that of the contralateral side (r_s_ = 0.4, 95 %-CI [0.26, 0.52], *p* < 0.001). The mean amount of CC (in % of total cartilage area) of the MH correlated significantly with the severity of hOA (r_s_ = 0.51, 95 %-CI [0.32, 0.65], *p* < 0.001). SEM revealed significant associations between CC and hOA (r = 0.74, *p* < 0.001) and between hOA and age (β = 0.62, *p* = 0.001), but not between CC and age (*p* = 0.15). There was no significant influence of BMI on either CC (*p* = 0.37) or hOA (*p* = 0.16).

**Conclusion:**

The observation that CC of the 1^st^MH is significantly associated with the severity of OA but independent of age and BMI, suggests an intimate relationship between CC and the pathogenesis of OA, the exact nature of which will have to be explored by future studies.

**Electronic supplementary material:**

The online version of this article (doi:10.1186/s12891-016-1324-0) contains supplementary material, which is available to authorized users.

## Background

Foot pain is a frequent clinical concern. One reason for foot pain is osteoarthritis (OA) [1–3], in particular of the first metatarsophalangeal joint (1^st^ MTPJ) [4, 5]. The prevalence of radiographic OA of the 1^st^ MTPJ in middle-aged and older adults in the general population has been described to vary between 12 and 42.4 % [6–8]. Although there are risk factors for OA of the 1^st^ MTPJ, namely anatomic variations [4, 9–11], altered biomechanics [4, 12, 13], trauma [14–16] and metabolic or chronic inflammatory disorders such as rheumatoid arthritis and gout [17, 18], until today, the etiology of OA of the 1^st^ MTPJ remains unknown in the majority of cases.

Tissue calcification in humans is a complex but incompletely understood process and occurs exclusively in hard tissues (bone, growth plate, teeth) under physiological conditions [19]. Any deposition of mineral calcium-phosphate compounds outside these hard tissues is considered to be a pathological process and is called ectopic calcification [20]. The calcification of articular cartilage is also known as chondrocalcinosis [21].

The visualization of articular cartilage calcification is technically difficult and the sensitivity of detection strongly depends on the applied imaging method [22, 23]. Until today, published data of joint calcification at various sites (knee, hip, hand, wrist and symphysis pubis) [24–30] is mostly based on low-resolution imaging techniques, i.e. standard X-ray, CT or MRI. The detection of hyaline cartilage calcification with these methods is insufficient for the visualization of small initial calcification stages [31–33]. The reported prevalence of articular cartilage calcification in the literature is almost exclusively based on plain radiographs and therefore is likely to underestimate the real prevalence [34, 35]. The most sensitive method to detect cartilage calcification is digital contact radiography (DCR) [23]. Work from our group has previously demonstrated deposition of cartilage calcification in 100 % of severely osteoarthritic hip and knee joints [22, 31]. Since in these studies there was a significant correlation between the amount of cartilage calcification and the clinical severity of hip and knee OA [22, 31], the clinical relevance of cartilage calcification in OA appears to be evident. Nonetheless, the relationship between cartilage calcification and OA is not fully understood, nor is the interrelationship between age, OA and cartilage calcification. In particular, it remains unclear whether cartilage calcification represents a simple epiphenomenon of ageing or whether there is a causal relationship between cartilage calcification and OA [36].

Regarding the 1^st^ MTPJ, only periarticular but not intra-articular calcification has been visualized and described so far [37–39]. To the best of our knowledge, there is no data in the literature about calcification of the articular hyaline cartilage of the 1^st^ MTPJ.

Here we analyzed for the first time the prevalence of articular cartilage calcification of the 1^st^ MTPJ in a post-mortem cohort of the general population by DCR and examined the relationship between cartilage calcification and histological OA grade, age and BMI.

## Methods

This cross-sectional study was approved by the local Ethics Committee of the Medical Association Hamburg, Germany (Ärztekammer Hamburg, PV: 4570) and was carried out according to existing rules and regulations of the University Medical Center Hamburg-Eppendorf. 168 first metatarsophalangeal joints (1^st^ MTPJ) were obtained from 84 donors at the Department of Legal Medicine, University Medical Center Hamburg-Eppendorf. Only donors with intact metatarsophalangeal joints without any signs of disease other than OA were included in this study. Donors, which had evidence for previous surgical intervention or fractures of the metatarsophalangeal joint, or any rheumatic or oncologic disease were excluded from the study population.

### Sample preparation

After removal of soft tissue a standardized 4 mm cartilage-bone slab was cut in the central axial plane from each metatarsal head (MH) (Additional file 1: Figure S1). To avoid potential contamination with sawing dust the slabs were cleaned in physiological saline solution and the residual bone debris was removed.

### Digital contact radiography (DCR)

Standardized radiographs (25 kV, 3.8 mAs, film focus distance 8 cm) were taken from each cartilage-bone slab of the MH using a high-resolution digital radiography device (Faxitron X-Ray, Illinois, USA). In these radiographs, calcifications of the hyaline cartilage could be detected as radiopaque spots within the surrounding cartilage matrix. The mean amount of total cartilage calcification in % of total cartilage area for each MH was measured by image-analysis software (ImageJ 1.46, National Institutes of Health, Bethesda, USA) as described previously [22, 40].

### Histological examination

Histological assessment was performed of the main load-bearing zone of each MH (central zone, directly adjacent to the central axial slab plane). A specimen of full thickness hyaline cartilage was cut to the subchondral bone plate of the load-bearing zone.

All specimens were fixed in 4 % PFA for 24 h, dehydrated in 80 % alcohol and embedded in paraffin. Four micrometer sections were cut and stained as shown in Additional file 2: Figure S2 with

1. Safranin Orange (1 %) - to evaluate the histological OA grade according to the osteoarthritis cartilage histopathology assessment system (grade 0–6) (OARSI) [41].
2. von Kossa (counterstained with Light Green) - to confirm the deposition of calcium-phosphate-crystals in the hyaline cartilage.

### Statistical analysis

The biometric characteristics of donors are reported as mean values ± standard deviations (SD). For descriptive analysis, mean cartilage calcification values for each side were used. Fisher’s and McNemar’s exact test were used for categorical data. Spearman’s rank correlation coefficient was calculated to report associations between continuous variables. To analyze the correlation between the mean amount of cartilage calcification and histological degeneration grade, respectively age, the mean value of the left and right metatarsal head (MH) were calculated for each person. Structural equation modeling (SEM) [42] was used to investigate relationships between cartilage calcification, histological OA grade, age and BMI. The histological OA grade and cartilage calcification were included as latent variables, age and BMI as manifest variables. SEM accounts for intraclass correlation and allows to combine factor, multiple regression and covariance analysis simultaneously to describe interrelated relationships, as well as the representation of unobserved theoretical constructs (latent variables), while directly accounting for measurement error in the estimation [43]. Diagonally weighted least squares (DWLS) estimation was used. To assess model fit Chi-squared statistic and Root Mean Square Error of Approximation (RMSEA) was calculated. Cartilage calcification was transformed binary (0 = negative and 1 = positive DCR-detectable cartilage calcification of the 1^st^ MH). All statistical analyses were performed with statistical software R (version 3.1.1) [44], and the package lavaan [45] and ggplot2 [46]. Regression coefficient β and correlation coefficient r were reported. A *p*-value less than 0.05 was considered statistically significant.

## Results

In this study 168 metatarsal heads (MH) from 84 individual donors (*n* = 37 female and *n* = 47 male) were analyzed by DCR and histological assessment. The mean age was 62.7 years (SD ±18.8, range 20–93); 37 of the donors were female and 47 male. The distribution of gender and age by decade is shown in Additional file 3: Figure S3. The biometric characteristics of the study population are listed in Table 1.

**Table 1:** Biometric characteristics of the study population (*n* = 84; 37 female and 47 male)

	Mean	SD
Age in years	62.73	18.80
Female	65.89	18.39
Male	60.23	18.93
Height in cm	171.92	8.38
Female	166.14	6.25
Male	176.5	6.9
Body weight in kg	78.68	19.46
Female	74.59	20.21
Male	81.7	18.4
body mass index (BMI) in kg/m^2^	26.54	6.00
Female	26.98	6.67
Male	26.2	5.5

The biometric characteristics are reported as mean values and standard deviations (SD) of the mean values

### Prevalence of cartilage calcification of the 1^st^ MH analyzed by DCR

The prevalence of hyaline cartilage calcification of the 1^st^ MH in the study population was 48.8 % (41 of 84 donors) (95 %-CI [37.7 %, 60.0 %]) (Table 2). The left joint was affected in 31.0 % (26/84) (95 %-CI [21.3 %, 42.0 %]) and the right joint in 36.9 % (31/84) (95 %-CI [26.6 %, 48.1 %]), no preference for cartilage calcification of one side could be detected (*p* = 0.42). Unilateral cartilage calcification was observed in 61.0 % (95 %-CI [44.5 %, 75.8 %]) of the affected donors (25/41) and bilateral calcification in 39.0 % (16/41) (95 %-CI [24.2 %, 55.5 %]). There was a significant correlation between the mean amount of cartilage calcification of one MH with the mean amount of cartilage calcification of the contralateral side in individuals with bilateral calcification (r_s_ = 0.40; 95 %-CI: [0.26, 0.52], *p* < 0.001). Overall, cartilage calcification was detectable in 57 of 168 joints (33.9 %).

**Table 2:** Prevalence of DCR-detectable hyaline cartilage calcification (CC) of the first MH in the study population

	n	in %	95 %-CI
Calcification	41/84	48.8	37.7 %, 60.0 %
Female	19/37	51.4	34.4 %, 68.1 %
Male	22/47	46.8	32.1 %, 61.9 %
Unilateral calcification	25/41	61.0	44.5 %, 75.8 %
Female	10/19	52.6	28.9 %, 75.6 %
Male	15/22	68.2	45.1 %, 86.1 %
Bilateral calcification	16/41	39.0	24.2 %, 55.5 %
Female	9/19	47.4	24.4 %, 71.1 %
Male	7/22	31.8	13.9 %, 54.9 %

Histochemical staining with von Kossa confirmed the presence of hyaline cartilage calcifications (Additional file 2: Figure S2 B).

The distribution of the prevalence of cartilage calcification by age and BMI is shown in Table 3.

**Table 3:** Prevalence distribution of cartilage calcification of the first MH by age and BMI

Age	n	in %	95 %-CI
<30	1	33.3	0.8 %, 90.6 %
<40	6	54.5	23.4 %, 83.3 %
<50	8	36.4	17.2 %, 59.3 %
<60	14	41.2	24.6 %, 59.3 %
<70	20	42.6	28.3 %, 57.8 %
<80	28	43.1	30.8 %, 56.0 %
<90	38	47.5	36.2 %, 59.0 %
≥90	41	48.8	37.7 %, 60.0 %
BMI	n	in %	95 %-CI
<20	9	69.2	38.6 %, 90.9 %
<25	18	54.5	36.4 %, 71.9 %
<30	34	52.3	39.5 %, 64.9 %
<35	39	50.0	38.5 %, 61.5 %
<40	40	49.4	38.1 %, 60.7 %
≥40	41	49.4	38.2 %, 60.6 %

### Gender-specific prevalence of cartilage calcification of the 1^st^ MH

Cartilage calcification in at least one MH was detected by DCR in 51.4 % of female (19/37) (95 %-CI [34.4 %, 68.1 %]) and in 46.8 % of male donors (22/47) (95 %-CI [32.1 %, 61.9 %]), corresponding to an overall prevalence of cartilage calcification within the study population of 48.8 % (95 %-CI [37.7 %, 60.0 %]) (Table 2). In summary, no significant difference in cartilage calcification of the 1^st^ MH could be detected for gender (*p* = 0.41).

### Correlation of cartilage calcification of the 1^st^ MH with histological OA grade by OARSI

The distribution of the histological OA grade by OARSI and the corresponding prevalence of DCR-detectable cartilage calcification is shown in Table 4 (*n* = 168). A histological OA grade by OARSI ≥ 3 was considered as severe osteoarthritis.

**Table 4:** Distribution of the histological OA grade and the corresponding prevalence of cartilage calcification of the 1^st^ MH (n = 168)ᅟ

hOA by OARSI	Distribution of hOA		DCR-detectable cartilage calcification	
(0–6)	%	(n)	%	(n)
0	12.5	21/168	14.3	3/21
1	54.2	91/168	24.2	22/91
2	19.0	32/168	43.8	14/32
3	8.3	14/168	64.3	9/14
4	2.4	4/168	100.0	4/4
5	2.4	4/168	75.0	3/4
6	1.2	2/168	100.0	2/2
Grade < 3	85.7	144/168	27.1	39/144
Grade ≥ 3	14.3	24/168	75.0	18/24

Distribution of the histological OA grade (hOA) by OARSI (0–6) and the corresponding prevalence of DCR-detectable cartilage calcification of all analyzed metatarsal heads (*n* = 168) in the study population. A histological OA grade by OARSI ≥ 3 was considered as severe osteoarthritis

In 85.7 % (144/168) of all analyzed metatarsal heads only mild cartilage damage (OARSI < 3) was detected. Accordingly, 14.3 % (24/168) of all metatarsal heads displayed severe cartilage damage (OARSI ≥ 3). Cartilage calcification was detectable by DCR in 75 % of samples with OARSI ≥ 3 (18/24). Interestingly, even in the samples with mild or no cartilage damage (OARSI < 3) cartilage calcification was also detectable in 27.1 % (39/144) of the analyzed samples.

**Fig. 1:**
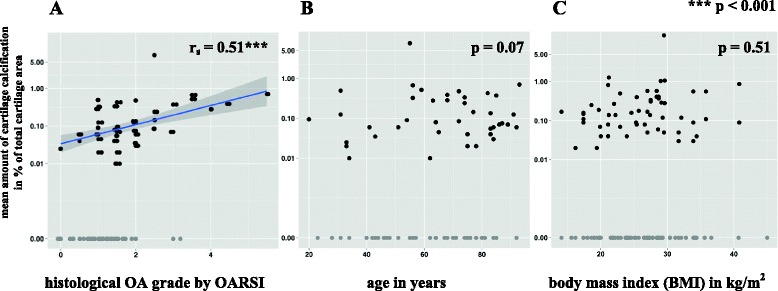
Correlation between CC and histological OA grade by OARSI (**a**), between CC and age (**b**) and between CC and BMI (**c**). The scatter diagrams (data points are jittered to avoid overplotting) show the relation between the mean amount of cartilage calcification (in % of total cartilage area) and histological OA grade by OARSI (**a**), respectively age in years (**b**) and BMI (**c**). For better overview, the y-axis is displayed on a logarithmic scale. The scatter diagram A is shown with an orthogonal linear regression line (blue line). The mean amount of cartilage calcification correlated significantly with the histological OA grade (**a**) (r_s_ = 0.51, 95 %-CI[0.32, 0.65], *p* < 0.001) but not with age (**b**) (r_s_ = 0.20, 95 %-CI[−0.02, 0.40], *p* = 0.07) or BMI (**c**) (r_s_ = −0.07, 95 %-CI[−0.17, 0.14], *p* = 0.51)

In addition, the percentage of samples with DCR-detectable cartilage calcifications rises with increase of the histological OA grade by OARSI (Table 4) and there was a significant correlation between the mean amount of cartilage calcification (in % of total cartilage area) of the MH and histological OA grade by OARSI (r_s_ = 0.51, 95 %-CI [0.32, 0.65], *p* < 0.001) (Fig. 1a).

### Correlation of cartilage calcification of the 1^st^ MH and age

There was no significant correlation between the mean amount of cartilage calcification (in % of total cartilage area) with age (r_s_ = 0.20, 95 %-CI [−0.02, 0.40], *p* = 0.07) (Fig. 1b).

### Correlation of cartilage calcification of the 1^st^ MH and BMI

There was no significant correlation between BMI and the mean amount of hyaline cartilage calcification. (Fig. 1c) (r_s_ = −0.07, *p* = 0.51).

### Interrelationship between cartilage calcification, histological OA grade, age and BMI

To analyze the interaction of cartilage calcification, histological OA grade, age and BMI simultaneously, simple regression analysis is not an appropriate tool. For a simultaneous analysis of these interrelationships we used structural equation modeling (SEM), which directly accounts for measurement error in the estimation. The histological OA grade and cartilage calcification were included as latent variables, age and BMI as manifest variables. Latent variables are factors, which consist of at least two inter-related measured variables. They are called latent because they are not directly measured, but rather are represented by the overlapping variance of measured variables. They are said to better represent the research constructs than measured variables because they contain less measurement error [47].

**Fig. 2:**
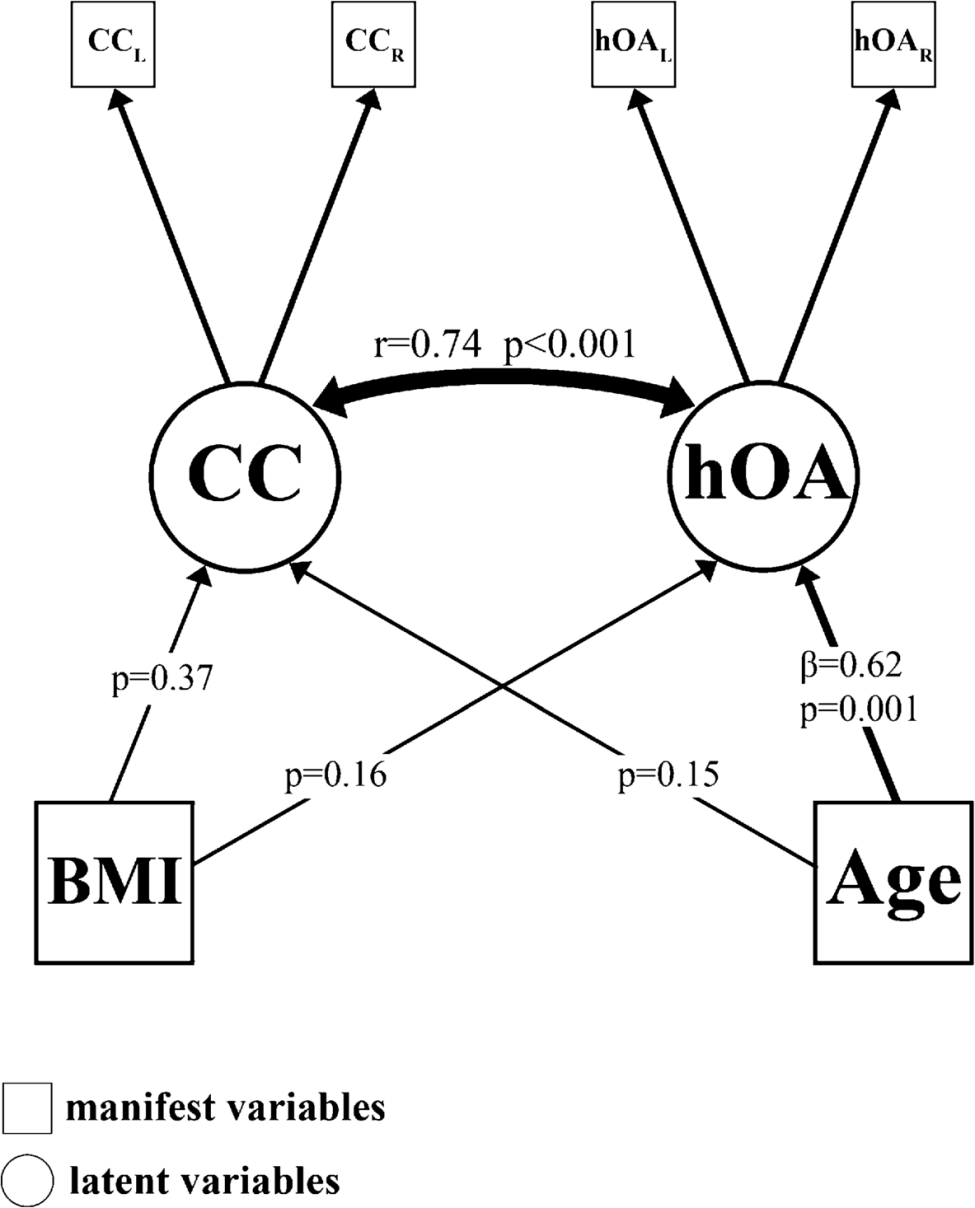
Structural equation model. Manifest (measured) variables are represented by boxes, latent (i. e. not observable) variables are represented by circles. *Abbreviations:* hOA = histological osteoarthritis grade, CC = cartilage calcification, Age = age in years, BMI = body mass index [kg/m2], CCL/R = DCR-detctable hyaline cartilage calcification of the left/right central axial slab of MH, OAL/R = histological OA grade (by OARSI) of the left/right MH

The SEM is schematically represented in Fig. 2. There was a significant correlation between cartilage calcification and histological OA grade (r = 0.74, 95 %-CI [0.43, 1.05], *p* < 0.001). Age had no influence on cartilage calcification (*p* = 0.15, 95 %-CI [−0.08, 0.53]), but a significant influence on histological OA grade (β = 0.62, 95 %-CI [0.26, 0.99], *p* = 0.001). BMI had no influence on cartilage calcification (*p* = 0.37, 95 %-CI [−0.45, 0.17]) or histological OA grade (*p* = 0.16, 95 %-CI [−0.56, 0.09]). The measures of global model fit indicated a good model fit (Chi-square = 7.83, *p* = 0.17, df = 5; RMSEA = 0.08).

## Discussion

In this cross-sectional study we analyzed and quantified articular cartilage calcification of 168 metatarsophalangeal joints by a high-resolution imaging technique (DCR) in a post-mortem cohort of 84 adult donors. There prevalence of cartilage calcification of the 1^st^ MTPJ in the cohort was 48.8 % (41 of 84 donors). Cartilage calcification was detectable in 33.9 % of all analyzed joints (57/168). In cases with bilateral manifestation, there was a significant correlation between the mean amounts of cartilage calcification of one joint with the mean amount of cartilage calcification of the contralateral side. The mean amount of cartilage calcification correlated significantly with histological OA grade, but not with age, gender or BMI.

To our knowledge, this is the first time that articular cartilage calcification of the first MTP joint is described and quantified by a high-resolution imaging technique (DCR).

Other cross-sectional studies have analyzed the prevalence of cartilage calcification by standard radiography in various joints. The reported prevalence of CC was considerably lower than in the current study (knee 7–21 %, hip 0,4–5 %, wrist 5–8 % and symphysis pubis 3–12 %) [24–30]. The only study that has applied DCR to detect cartilage calcification in the general population has been published by Mitsuyama et al. who have observed cartilage calcification in 100 % of 106 knee joints from the general population (mean age 50.3 years, range 12–74) [40]. In this paper, the authors have detected cartilage calcification already in knees of young donors (<20 years) without macroscopically severe OA and they have shown an association between increasing amount of cartilage calcification and increasing age [40], but did not adjust for the histological OA grade in their analyses. In our study, hyaline cartilage calcification was also detected in joints of young donors, but in contrast to Mitsuyama et al., we adjusted for histological OA grade and did not find an association between cartilage calcification and age. It is conceivable, although currently speculative, that the association of knee joint CC with age in the study of Mitsuyama et al. would disappear after adjustment for OA grade. Based on our current data, at least in the 1^st^ MTPJ, we believe that cartilage calcification is an age-independent process. In the heavily loaded 1^st^ MTPJ, mechanical stress could be a predisposing factor for CC. However, in the present analysis, the additional mechanical stress induced by increasing BMI did not appear to have an influence on cartilage calcification, nor on OA grade of the 1^st^ MTPJ. These data argue against mechanical load as a relevant contributing factor in the genesis of cartilage calcifications. Along similar lines, Nguyen et al. described that hyaline cartilage calcification was also found in less weight-bearing zones of knee joints [48]. To clarify the relevance of these still preliminary observations, further studies with direct comparisons of weight-bearing and non-weight-bearing joints, respectively joints zones are required.

The observation that cartilage calcification is already observed to a large degree in samples without signs of severe OA (CC in 27.1 % of all analyzed samples with OARSI < 3) is highly intriguing. Similar findings have been described in two animal models of spontaneous OA, in which cartilage calcification was detected before cartilage degeneration occurred [49, 50]. Taken together, these data confirm the notion that cartilage calcification occurs not only in OA joints but also in histologically intact hyaline cartilage. Although this is strongly suggestive of a causal relationship between CC and OA, the biological meaning of this observation will have to be uncovered by future studies.

Nevertheless and as expected from observations in other joints, we also found a significant relationship between cartilage calcification of the 1^st^ MTPJ and histological OA grade. Taken together, these data can be seen as a hint that increasing amount of cartilage calcification may trigger and accelerate OA progression by yet to be identified molecular mechanisms.

This study has some limitations. There was no detailed history of the donors about predisposing factors of OA of the 1^st^ MTPJ such as infection, previous trauma or chronic mechanical overload. Moreover, it remains unclear whether the donors had clinical symptoms such as pain or reduced mobility of the 1^st^ MTPJ. Another limitation of the study was that the analyzed cartilage-bone slabs of the metatarsal head reflect only a small part of the whole cartilage surface of the joint. Finally we have not performed an analysis of the type of cartilage calcification (BCP or CPPD). For this characterization a complex and time-consuming analysis by FTIR spectroscopy or X-Ray diffractometry is required, which was not the objective of this study.

## Conclusion

DCR analysis of articular cartilage calcification of the 1^st^ MTPJ in a cross sectional cohort of the general population revealed that cartilage calcification already occurs in histologically intact hyaline cartilage, is associated with OA severity but is independent of age and BMI.

These novel and partially unexpected data are suggestive of an intimate relationship between cartilage calcification and the pathogenesis of OA but at the same time demonstrate that a lot more work is required before we will reach a comprehensive understanding of the biological and pathophysiological role of hyaline cartilage calcification.

## Additional files

Additional file 1: Figure S1. Sample preparation and DCR. **A**. Each metatarsal head (MH) of the 1st MTPJ was cut in one standardized 4 mm thick cartilage-bone-slab (dashed line) along the central axial plane. **B**. Radiographs were taken from each cartilage-bone slab of the MH using a high-resolution digital radiography device (DCR). In these radiographs, calcifications of the hyaline cartilage could be detected as radiopaque spots (red arrow) within the surrounding cartilage matrix. The mean amount of total cartilage calcification in % of total cartilage area for each MH was measured by image-analysis software. (DOCX 6230 KB)
Additional file 2: Figure S2. DCR-images and histological examination. **A**. DCR-images with hyaline cartilage calcifications (original size and 3 × magnification) of cartilage-bone slabs of the metatarsal heads from different donors and the corresponding Safranin O stainings from the central load-bearing zone of the metatarsal head. Histological OA grade was evaluated by the OARSI score. Deposition of cartilage calcification was detectable in all OA grades (OARSI 0–6) by DCR. **B**. The existence of DCR-detectable hyaline cartilage calcification (calcium-phosphate-crystals) was histochemically confirmed by von Kossa stainings. (DOCX 2250 KB)
Additional file 3: Figure S3. Study population histogram by decade. 84 donors; 37 female and 47 male. The mean age was 62.73 years, SD ± 18.8, range 20–93 years. (DOCX 20 KB)

## Abbreviations

1^st^MTPJ: First metatarsophalangeal joint
BMI: Body mass index
CC: Cartilage calcification
DCR: Digital contact radiography
hOA: Histological osteoarthritis grade
MH: Metatarsal head
OA: Osteoarthritis
OARSI: Osteoarthritis cartilage histopathology assessment system
SEM: Structural equiation modeling
