# Supplementary figures and images for: Hyaline cartilage calcification of the first metatarsophalangeal joint is associated with osteoarthritis but independent of age and BMI
Source: BMC Musculoskelet Disord. 2016 Nov 15;17:474. doi: 10.1186/s12891-016-1324-0 (PMC5109667; doi:10.1186/s12891-016-1324-0)

| **Figure S1.** |
| --- |
| 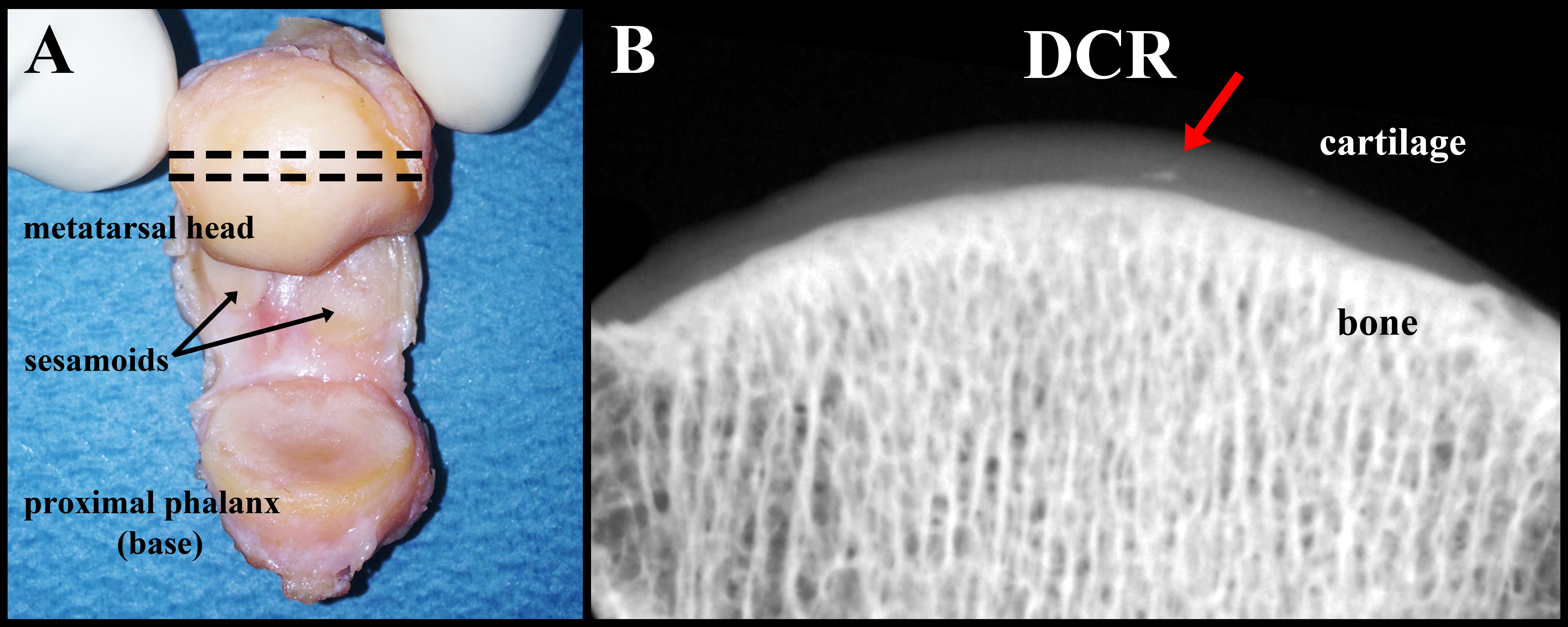 |
|  |

Supplement: Additional file 1: Figure S1. — Sample preparation and DCR. A. Each metatarsal head (MH) of the 1st MTPJ was cut in one standardized 4 mm thick cartilage-bone-slab (dashed line) along the central axial plane. B. Radiographs were taken from each cartilage-bone slab of the MH using a high-resolution digital radiography device (DCR). In these radiographs, calcifications of the hyaline cartilage could be detected as radiopaque spots (red arrow) within the surrounding cartilage matrix. The mean amount of total cartilage calcification in % of total cartilage area for each MH was measured by image-analysis software. (DOCX 6230 KB) [file 12891_2016_1324_MOESM1_ESM.docx]

| **Figure S2.** |
| --- |
| 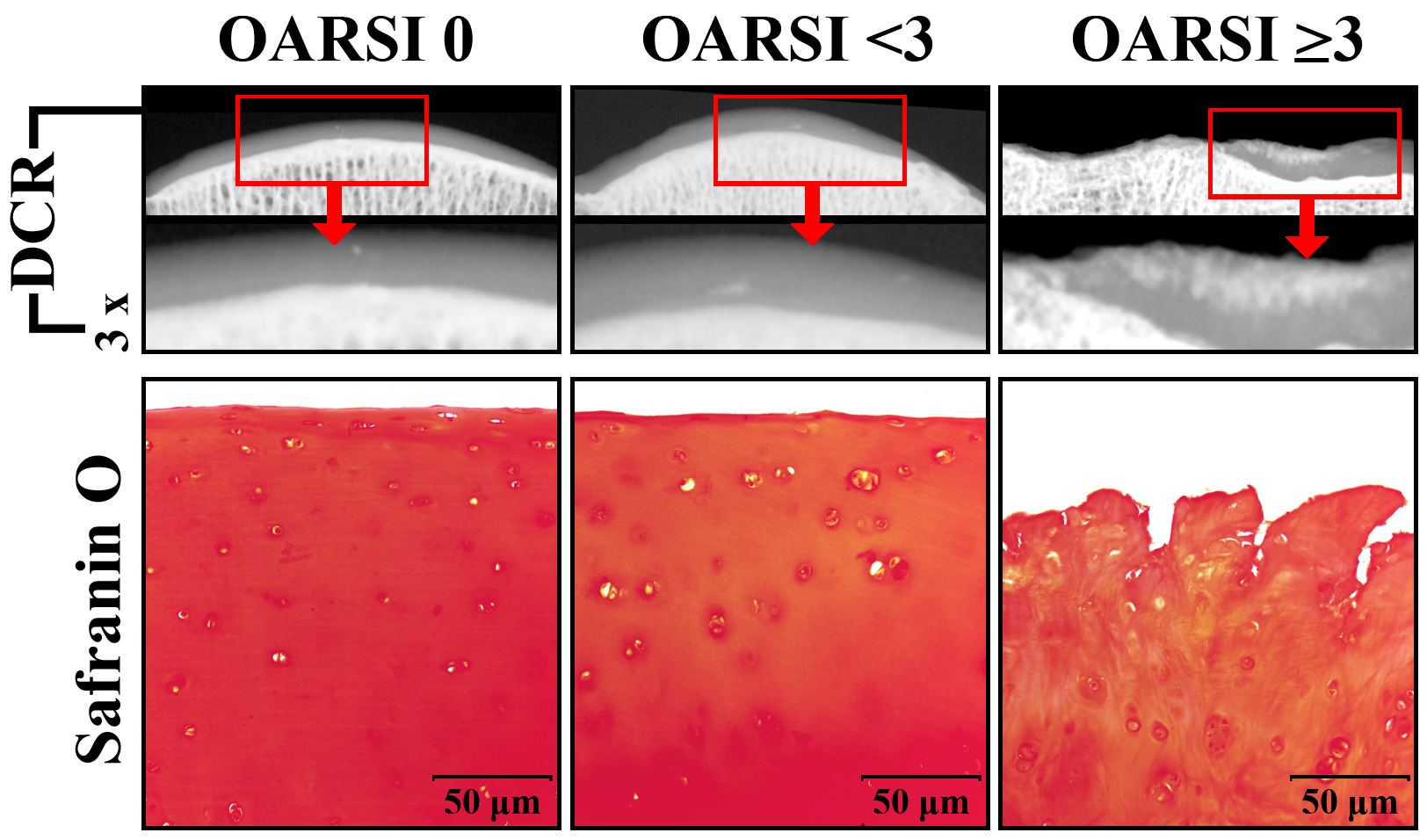  **A** |
| **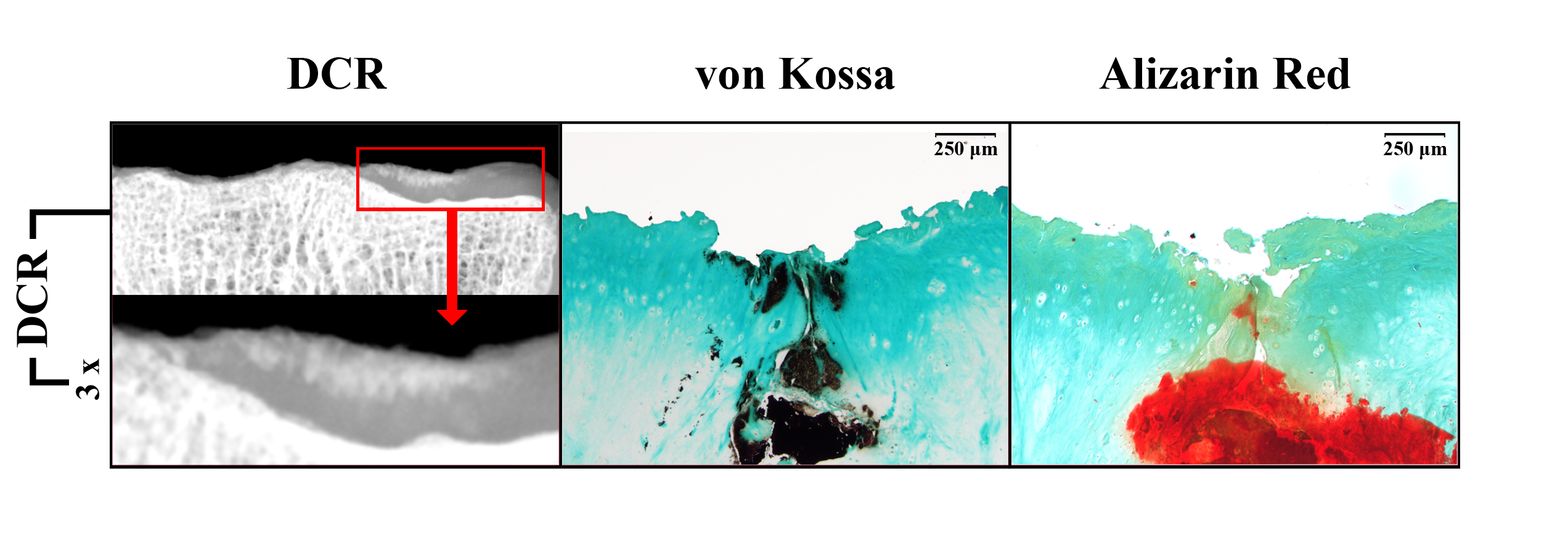**  **B** |
|  |

Supplement: Additional file 2: Figure S2. — DCR-images and histological examination. A. DCR-images with hyaline cartilage calcifications (original size and 3 × magnification) of cartilage-bone slabs of the metatarsal heads from different donors and the corresponding Safranin O stainings from the central load-bearing zone of the metatarsal head. Histological OA grade was evaluated by the OARSI score. Deposition of cartilage calcification was detectable in all OA grades (OARSI 0–6) by DCR. B. The existence of DCR-detectable hyaline cartilage calcification (calcium-phosphate-crystals) was histochemically confirmed by von Kossa stainings. (DOCX 2250 KB) [file 12891_2016_1324_MOESM2_ESM.docx]
