# Supplementary material for: Hyaline cartilage calcification of the first metatarsophalangeal joint is associated with osteoarthritis but independent of age and BMI
Source: BMC Musculoskelet Disord. 2016 Nov 15;17:474. doi: 10.1186/s12891-016-1324-0 (PMC5109667; doi:10.1186/s12891-016-1324-0)
Supplement: Additional file 3: Figure S3. — Study population histogram by decade. 84 donors; 37 female and 47 male. The mean age was 62.73 years, SD ± 18.8, range 20–93 years. (DOCX 20 KB) [file 12891_2016_1324_MOESM3_ESM.docx]

| **Figure S3.** |
| --- |
|  |
|  |
